# Evaluation of fotonovela to increase human papillomavirus vaccine knowledge, attitudes, and intentions in a low-income Hispanic community

**DOI:** 10.1186/s13104-015-1609-7

**Published:** 2015-10-29

**Authors:** Alvin Chan, Brandon Brown, Enedina Sepulveda, Lorena Teran-Clayton

**Affiliations:** School of Medicine, University of California, Irvine, Irvine, CA USA; Center for Healthy Communities, School of Medicine, University of California, Riverside, Riverside, CA USA; Department of Population Health and Disease Prevention, Program in Public Health, University of California, Irvine, Irvine, CA USA; Clayton MD Total Health Center, San Diego, CA USA

**Keywords:** HPV vaccine, Fotonovela, Health literacy, Health education, Educational intervention, Narrative, Latino/Hispanic

## Abstract

**Background:**

It has nearly been a decade since the introduction of the vaccine against human papillomavirus (HPV), yet vaccination rates in the United States have remained suboptimal, particularly among Hispanics. Culturally and linguistically relevant health education tools targeting Hispanics are needed to increase the current rate of HPV vaccination. This article evaluates a theory-informed, evidence-guided fotonovela (photographic short story) intervention to improve HPV vaccination knowledge, attitudes, and intention among young adults.

**Methods:**

Young adults (N = 41, aged 18–26 years) in a low-income primary care clinic in Southern California were administered pre- and post-intervention surveys to measure changes in perceived susceptibility to HPV, perceived benefit of vaccination in committed relationship, intention to vaccinate, intention to encourage social networks to vaccinate, and attitude towards vaccination. Post-intervention survey also examined attitudes towards fotonovela. Relationships between attitudes towards fotonovela and demographic characteristics were assessed with Fisher’s exact test. Self-reported gains in knowledge were categorized and tabulated. Changes in perceptions and intentions were analyzed with the marginal homogeneity test.

**Results:**

The majority of participants were female (78.0 %), Latino/Hispanic (92.7 %), single (70.7 %), and had at least a college education (61.0 %). The mean age was 21.9 years (SD 0.4). The fotonovela was viewed as entertaining (95.1 %), educational (97.6 %), and easy to read (100 %). Following the intervention, Hispanic participants improved in all five variables of interest measured in the survey, including perceived susceptibility (+10.5 %, *p* = 0.03), benefit of vaccination (+7.8 %, *p* = 0.25), intent to vaccinate (+18.4 %, *p* = 0.06), intent to encourage others to vaccinate (+10.5 %, *p* = 0.14) and attitude towards vaccination (+13.1 %, *p* = 0.05). Improvements in perceived susceptibility and attitude towards vaccination reached statistical significance (p < 0.05). The most frequent gains in knowledge were the risk of HPV infection despite condom use (N = 16) and relationship status (N = 8), three-dose vaccine administration schedule (N = 13), and burden of HPV infection among males (N = 9).

**Conclusion:**

Results are promising because they demonstrate that health messages delivered through a narrative format can promote positive changes in knowledge, attitudes, and intentions. The fotonovela may be a powerful vehicle for HPV education, particularly among Hispanics.

## Background

Approximately 14 million Americans are newly infected by human papillomavirus (HPV) each year, making it the most common sexually transmitted infection in the United States [1]. While most sexually active individuals are exposed to HPV, the majority clear the infection without symptoms [2–4]. However, for those who have persistent infection, the virus may cause precancerous lesions that, if untreated, could progress to cervical cancer as well as anal, penile, vaginal, vulvar, and oropharyngeal cancers [5–7]. Although potentially lifesaving vaccines exist to protect against HPV-related cancers [8, 9], vaccination rates remain low, particularly among Hispanics who already suffer from disproportionately high rates of HPV-related disease [10–12]. Hispanic women are diagnosed with cervical cancer at 1.4-times the rate of non-Hispanics [13]. Likewise, Hispanic men have 1.7-times the incidence rate of HPV-related penile cancer of non-Hispanic males [14].

Lack of knowledge regarding HPV and HPV vaccination may contribute to the high rate of non-vaccination among Hispanics [15–17], in part because they do not know enough about HPV to make informed decisions about vaccination [12, 18–21]. The US Department of Health & Human Sciences reports that 65 % of Hispanic adults have below basic to basic health literacy skills, which may not be sufficient to understand most health materials [22]. Common gaps in knowledge include risk factors for HPV [21, 23, 24], link between HPV and cancer [25], necessity of vaccination for males [12, 25], and necessity of vaccination for individuals in committed relationships [25].

Although Hispanics are a priority group for HPV prevention, few educational interventions have been targeted specifically to this population. In a systematic review that examined resources designed to improve HPV vaccine knowledge and acceptance, 33 studies were evaluated and only a single study was culturally adapted to Hispanics [26]. This study by Kepka et al. tested the effectiveness of a Spanish radionovela, or broadcast short story, to educate rural Hispanic parents about HPV vaccination [27]; and although the intervention improved HPV knowledge, it was generally unsuccessful because there was no difference in intention to vaccinate one’s daughter between the experimental and control groups. In another study, Frank, et al. examined the effects of an 11-min English-language narrative film on perceived response efficacy, perceived severity and perceived susceptibility to HPV among European American, Mexican-American and African American women [28]. The investigators found that relevance of the storyline was particularly important for raising awareness about the severity of HPV and the effectiveness of the vaccine. Identification with specific characters was positively associated with perceptions of HPV susceptibility. Overall, the results underscored the importance of integrating narrative elements, namely storyline relevance and character identification, into health communication tools.

One type of intervention that shows promise as a health education tool in Spanish-speaking communities is the fotonovela [29–36]. Formatted with photographs and text bubbles similar to comic books, fotonovelas place educational messages within moving scripts that present characters and events that are familiar to Hispanic readers. Fotonovelas differ from traditional, non-narrative types of health materials in that they integrate social norms, depict positive role models, and incorporate dramatic storylines that deliver sensitive information in memorable ways [31]. Its storytelling approach also makes it an appropriate low-literacy educational medium when dialogue is written at a low reading level. In 2014, Boyte et al. developed a fotonovela that encourages Hispanic mothers to get their preteen children vaccinated against HPV [29]. The researchers demonstrated that the fotonovela could be a useful educational instrument to increase HPV vaccine knowledge among Hispanic mothers in three California communities.

In the present study, we addressed the gap in HPV vaccine uptake among Hispanics by creating a culturally and linguistically tailored educational intervention in the form of a fotonovela that aimed to increase HPV vaccine knowledge, attitudes, and intentions in young adults. We first presented the theory that informed the design of the fotonovela. Then, we described a quantitative summative evaluation of the fotonovela in a low-income Hispanic community in Southern California. We hypothesized that the fotonovela will not only improve knowledge, attitudes and intentions regarding vaccination, but also encourage dialogue about HPV prevention within the community.

## Methods

### Development of fotonovela

The Health Belief Model (HBM) was used to guide the design of the fotonovela because of its proven relevance to vaccination behavior [37–40]. The underlying concept of the HBM is that health behavior is determined by personal perceptions about a disease. Thus, to prompt individuals to get the HPV vaccine, specific HPV and HPV vaccine information were incorporated into the fotonovela to address the six constructs of the HBM, which included perceptions of susceptibility to disease, perceptions of severity of disease, perceptions of benefits of the health action, perceptions of barriers, self-efficacy, and cues to action.

Low perceived HPV susceptibility, severity, and benefits of vaccination were addressed by organizing educational messages around common knowledge gaps (risk factors for HPV, health consequences of HPV, link between HPV and cancer, benefits and efficacy of HPV vaccine for males and females) and misconceptions (safety of vaccine and side effects, belief that the vaccine is not necessary for males, belief that individuals in monogamous relationships are not at risk for contracting the virus). Self-efficacy was promoted through story protagonists, who served as role models for healthy behavior; it was believed that readers could learn from and emulate the characters’ actions. Lastly, the fotonovela itself served as a cue to action. Reading the story should motivate readers to initiate a dialogue about HPV prevention with their healthcare provider, family, and friends. Healthcare providers could reduce the perceived barriers of vaccine safety and cost through reassurance and referral to public programs that pay for the vaccine.

During a 6-month period in 2012–2013, the research team developed the script, photographed scenes, and designed the layout of the fotonovela. The fotonovela was piloted with community members prior to finalization to ensure cultural and linguistic appropriateness of plot, dialogue, characters, educational messaging, and visual content.

### Fotonovela

**Fig. 1:**
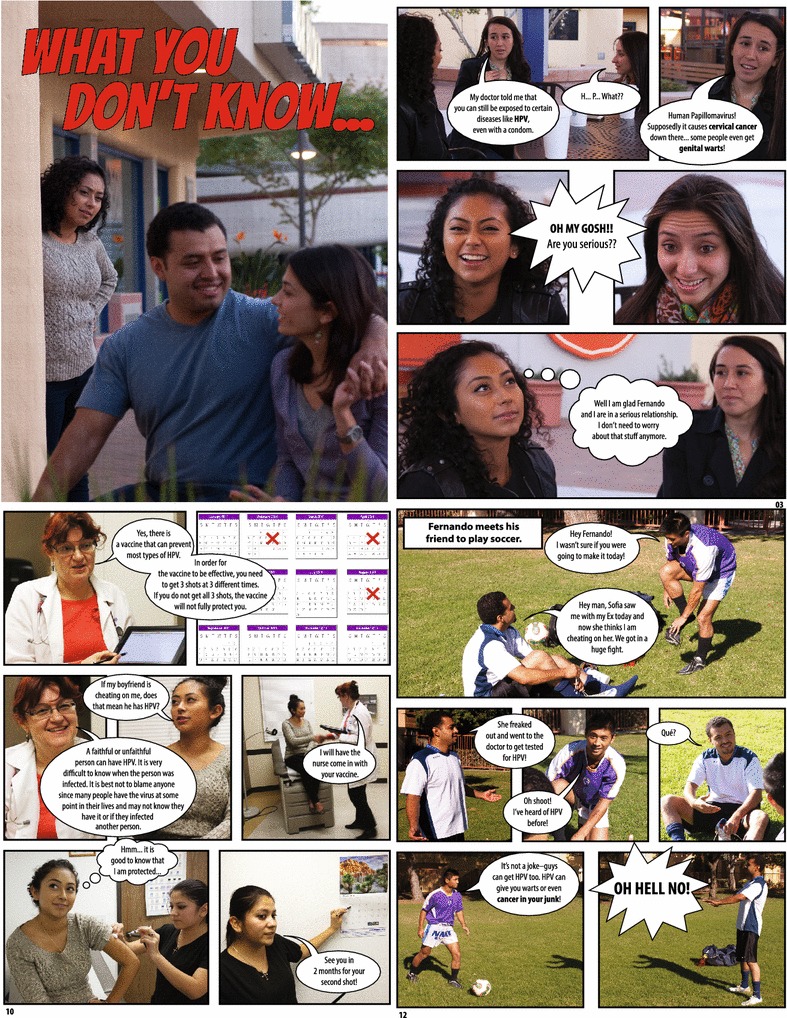
Sample scenes from fotonovela about the diseases associated with HPV infection, information about the HPV vaccine, and the male burden of HPV disease

The final product was an 18-page fotonovela entitled “What You Don’t Know” about a young Hispanic female who learned the importance of being vaccinated against HPV while in a committed relationship (Fig. 1). The fotonovela concluded with a “Question & Answer” section with key facts about HPV and the vaccine. The fotonovela was written in English at a sixth-grade reading level. A bilingual health care professional translated the fotonovela into Spanish. The English and Spanish versions of the fotonovela were pretested with a small set of community members prior to finalization.

### Participants

From September 2014 to February 2015, young adult participants from the University of California, Irvine Family Health Center (UCI-FHC) were recruited to participate in the study. UCI-FHC is a community-based, federally qualified health center in Southern California that serves a predominantly low-income Hispanic/Latino medically underserved population. Hispanics comprise 70 % of the overall clinic population, and 90 % of patients fall below 200 % of the federal poverty life. The majority of patients either lack insurance or have insurance through Medicaid, a federal- and state-funded social health care program for individuals with low income and limited resources, or Medical Services Initiative, a health care program for low-income citizens in Orange County, California. Male and female adult patients were recruited into the study if they were aged 18–26 years old.

### Procedures

Upon presentation to the clinic, participants were approached by our bilingual research personnel and asked to partake in a study about HPV. Participants who agreed to participate were screened for eligibility. After providing verbal informed consent, participants were handed a pretest survey, fotonovela, and posttest survey, in either Spanish or English. They were instructed to first complete the pretest survey, then read the fotonovela intervention, and subsequently complete the posttest survey. All questionnaires were anonymous in order to protect participant privacy. The study took place in the UCI-FHC waiting room and lasted 25 min. As compensation, participants were given the fotonovela. All study materials and procedures were approved by the UCI Institutional Review Board.

### Measures

This study used a pre-experimental study design to compare individual attitudes and intentions related to HPV and HPV vaccine pre- versus post-intervention. The questionnaire was divided into pretest (14 questions) and posttest Sects. (19 questions). The pretest section collected basic demographic information (9 questions) and preliminary attitudes and intentions towards HPV vaccination (5 questions). The same 5 variables of interest were administered again in the posttest section to assess for changes caused by the intervention (5 questions). The posttest section additionally evaluated attitudes about fotonovela content (13 questions) and gains in knowledge (1 question).

Attitudes and intentions towards HPV vaccination were assessed with 5 items on a 5-item Likert scale with an “Undecided” option. The items measured perceived susceptibility to HPV (“How likely are you to be infected with HPV?”), perceived benefit of vaccination in a committed relationship (“Is it necessary to vaccinate against HPV even if you are in a committed relationship?”), intention to vaccinate (“How likely will you get the HPV vaccine?”), intention to encourage family and friends to vaccinate (“How likely will you to encourage family and friends to get the HPV vaccine?”), and attitude towards vaccination (“How will getting the HPV vaccine make you feel”?).

The content of the fotonovela was assessed with 13 items on a dichotomous scale (yes/no). The items measured included entertainment value, educational quality, readability of text, cultural relevance, importance of presented information, relevance of information to personal life, familiarity of characters, and identification with characters. Additionally, there were items about willingness to disseminate the information with family and friends, willingness to share the fotonovela with family and friends, desire to learn more, and ability to use the learned information in everyday life. Gains in knowledge were evaluated with an open-ended question (“List 3 things that you learned after reading the booklet”). A research assistant, native in the source language and fluent in the target language, translated the questionnaire from English to Spanish. A second research assistant assessed the clarity, appropriateness of wording and acceptability of the translated questionnaire. The English and Spanish versions of the questionnaire were pretested prior to administration.

### Analyses

All quantitative analyses were performed using Stata 12.0 (Statacorp LP, College Station, TX, USA). Frequencies and percentages were computed for all variables. Due to small sample size, some responses answered on the Likert scale were collapsed for analyses: “Very likely,” “Likely,” and “Somewhat Likely” were combined into the category “Likely.” Responses about attitudes towards vaccination were also collapsed: “Empowered” and “Safe” were combined into the category “Positive” and “Anxious” and “Embarrassed” into “Negative.” Relationships between variables of interest and demographic characteristics of participants were assessed with Fisher’s exact test. Changes from pretest to posttest were analyzed with marginal homogeneity test. Self-reported gains in knowledge were analyzed for any patterns in responses and subsequently organized into distinct categories and tabulated. Since the purpose of this study was to develop a culturally and linguistically relevant health literacy tool targeting Hispanics, non-Hispanics (N = 3) were excluded from analyses and tables.

## Results

Of the 67 participants approached, 22 % refused to participate (N = 15), of which 80 % were men (N = 12). In total, 52 participants participated in the study, but 11 were excluded from analysis due to incomplete survey information. The 41 remaining participants were included in the analysis.

Most participants were female (78 %), Latino/Hispanic (92.7 %), single (70.7 %), and had at least a college education (61.0 %). As the majority of patients were born in the United States (73.2 %), most preferred to read in English (90.2 %) and approximately half spoke Spanish and English equally (53.7 %). Four participants (9.8 %) viewed the fotonovela and completed the questionnaires in Spanish. Hispanic participants with a higher education were more likely to have spent more years in the US (*p* < 0.05), to read in English (*p* < 0.02), and to speak in English (*p* < 0.01). The average age of participants was 21.9 years (SD 0.4). Participants under 21 years and those over 23 years were more likely to identify with the story characters (*p* < 0.05); no differences were found for other demographic characteristics with character identification (Table 1).

**Table 1:** Demographic characteristics of 38 Hispanic participants by identification with fotonovela characters

	All Hispanic participants, (N = 38), N (%)	Identified with characters, (N = 25), N (%)	Did not identify with characters, (N = 13), N (%)	Fisher’s *p* value for group differences
Age (years)				
18–20	12 (31.6)	9 (36.0)	3 (23.1)	0.046^a^
21–23	16 (42.1)	7 (28.0)	9 (69.2)	
24–26	10 (26.3)	9 (36.0)	1 (7.7)	
Sex				
Female	31 (81.6)	19 (76.0)	12 (92.3)	0.385
Male	7 (18.4)	6 (23.1)	1 (7.7)	
Country of birth				
United States	28 (73.7)	20 (80.0)	8 (61.5)	0.299
Mexico	9 (23.7)	5 (20.0)	4 (30.8)	
Other	1 (2.6)	0 (0)	1 (7.7)	
US residence (years)				
Born in US	28 (73.7)	20 (80.0)	8 (76.9)	0.440
1–5	2 (5.3)	1 (4.0)	1 (7.7)	
6–10	3 (7.9)	2 (8.0)	1 (7.7)	
>10	5 (13.2)	2 (8.0)	3 (15.4)	
Marital status				
Married	5 (13.2)	2 (8.0)	3 (23.1)	0.270
Living with partner	6 (15.8)	3 (12.0)	3 (23.1)	
Single	27 (71.1)	20 (80.0)	7 (53.8)	
Education				
Less than high school diploma	5 (13.2)	3 (12.0)	2 (15.4)	0.826
High school diploma or GED^b^	10 (26.3)	7 (28.0)	3 (23.1)	
Some college	17 (44.7)	12 (48.0)	5 (38.5)	
College degree	6 (15.8)	3 (12.0)	3 (23.1)	
Reading language preference				
English	34 (89.5)	22 (88.0)	12 (85.7)	1.00
Spanish	4 (10.5)	3 (12.0)	1 (7.7)	
Spoken language preference				
Only or mostly English	10 (26.3)	8 (32.0)	2 (15.4)	0.578
Only or mostly Spanish	6 (15.8)	4 (16.0)	2 (15.4)	
English and Spanish equally	22 (57.9)	13 (52.0)	9 (69.2)	

^a^Indicates statistical significance

^b^General Educational Development is considered equivalent to a high school diploma in the United States

Nearly all participants viewed the fotonovela as entertaining (95.1 %), educational (97.6 %), and easy to read (100 %). More than half identified with the characters (63.4 %) and related to the story (63.4 %). Most participants (95.1 %) agreed that the information conveyed in the fotonovela was important. Among them, 94.9 % said they would be able to use the information in their lives. Fisher’s exact test did not show any differences between Hispanics and non-Hispanics (*p* > 0.1 for all variables). Furthermore, there were no differences between participants who completed the study in Spanish compared to those who completed it in English (*p* > 0.5).

The majority of Hispanic participants (63 %) perceived the vaccine to be beneficial in committed relationships, intended to self-vaccinate and to encourage their friends and family to vaccinate, and had positive attitudes towards the vaccine at baseline. Only 21.1 % of Hispanic participants perceived themselves to be susceptible to HPV at baseline (Table 2). After the intervention, Hispanic participants were more likely to perceive susceptibility to HPV (+10.5 %, *p* = 0.03), to perceive benefit of vaccination in a committed relationship (+7.8 %, *p* = 0.25), to intend to vaccinate (+18.4 %, *p* = 0.06), to encourage others to vaccinate (+10.5 %, *p* = 0.14), and to have a positive attitude towards vaccination (+13.1 %, *p* = 0.05); however, only improvements in perceived susceptibility and attitude towards vaccination reached statistical significance. Hispanic participants in marriages or domestic partnerships reported higher susceptibility to HPV post-intervention compared to those who were single (*p* < 0.01). A positive attitude towards the HPV vaccine increased from 71.1 % at baseline to 84.2 % post-intervention (*p* < 0.05); and of the participants initially ambivalent towards the vaccine, 50 % later reported that they would feel safer with it. The only demographic characteristic significantly associated with intentions was age. Participants under 24-years old expressed a greater willingness to self-vaccinate (*p* = 0.02) and to encourage others to vaccinate (*p* = 0.02).

**Table 2:** Impact of fotonovela on perceptions and intentions related to HPV and HPV vaccine among 38 Hispanic participants

	Pretest			Posttest			*p* value^b^
	Likely, N (%)	Unlikely, N (%)	Undecided, N (%)	Likely, N (%)	Unlikely, N (%)	Undecided, N (%)	
Perceived susceptibility	8 (21.1)	20 (53.6)	10 (26.3)	12 (31.6)	24 (63.2)	2 (5.3)	0.034^a^
Perceived benefit of vaccination	30 (79.0)	1 (2.6)	7 (18.4)	33 (86.8)	0 (0)	5 (13.2)	0.247
Intent to vaccinate	24 (63.2)	7 (18.4)	7 (18.4)	31 (81.6)	4 (10.5)	3 (7.9)	0.060
Intent to encourage others to vaccinate	30 (79.0)	3 (7.9)	5 (13.2)	34 (89.5)	2 (5.3)	2 (5.3)	0.135

^a^Indicates statistical significance

^b^Marginal homogeneity test was used

In the free-response posttest question about knowledge gained, 83 % of participants reported multiple facts they had learned from the fotonovela. Participants most frequently listed the potential risk of HPV infection despite using condoms (N = 16) and being married or committed relationship (N = 8), the 3-dose HPV immunization schedule at 0, 2, and 6 months (N = 13), and the burden of HPV infection among males (N = 9) (Box 1). After reading the fotonovela, 85.4 % of participants planned to discuss the information with family and friends, and 95.1 % intended to share the fotonovela.

**Box 1:** Gains in knowledge at posttest listed by 33 participants, grouped into categories

Categories of knowledge gained	Number of times listed
I am at risk of HPV infection despite using condom	16
HPV vaccine is given as a series of three shots	13
HPV-related diseases are preventable with vaccine	12
Males are at risk for HPV infection	9
I am at risk of HPV infection even in a serious relationship	8
HPV is linked to cancer	5
It is possible to have HPV infection without knowing	3

## Discussion

Results from this study are promising because they demonstrate that health messages delivered through a narrative format can promote positive changes in knowledge, attitudes, and intentions. Specifically, the fotonovela increased people’s knowledge about the three-dose immunization schedule and the possibility of contracting HPV regardless of gender, relationship status and condom use. Additionally, the fotonovela improved people’s perceptions of their susceptibility to HPV, perceptions of the benefits of vaccination, attitude of vaccination, and intention to self-vaccinate and to prompt others to vaccinate.

Over 21 % of participants initially perceived themselves to being at risk of HPV. This coincides with previous studies that suggest between 21 and 46 % of adolescents and young adults believe they are at some risk of being infected with HPV [39]. Such low perceptions have been attributed to the uncertainly surrounding HPV transmission, particularly between partners in intimate relationships [25]. Thus, a major theme addressed in the fotonovela was the heightened risk of HPV infection in extra-relational affairs, a theme not discussed previously in the HPV radionovela, short film, and fotonovela interventions [27–29]. By underscoring the importance of vaccination in relationships, the story emphasizes the point that all sexually active individuals are susceptible to HPV-related disease, regardless of sexual history, relationship status, condom use and gender.

After the fotonovela intervention, perceived susceptibility increased. Also, participants who were married or involved in a domestic partnership reported significantly higher susceptibility than those who were single. Overall, these findings are encouraging because studies have shown that higher perceived susceptibility to HPV infection is related to higher vaccine acceptability [39, 40].

While the effectiveness of educational interventions on HPV vaccine acceptability has not been evaluated in the Hispanic young adult population, our study led to changes in vaccination intention that are comparable to research with other populations. In a Hong Kong study involving a 1-h educational slide presentation, the authors reported an 11.3 % increase in intention to vaccinate among Chinese adolescent girls [41]. Another study from the United Kingdom showed that 7 % more girls wanted to receive the vaccine after viewing a 10-min instructional video about HPV compared to those who did not [42]. Our fotonovela intervention resulted in a 19 % improvement in vaccination intention from 63 % at baseline to 82 % post-intervention among Hispanic young adults.

Although it is reassuring that 82 % of the people intended to vaccinate after reading the fotonovela, intentions have not historically translated to uptake within the Hispanic population. Previous studies have demonstrated similar acceptance rates among Hispanic parents, ranging from 73 to 97 % [43–45], yet the 3-dose HPV vaccine coverage is only 12.0 % among adolescent Hispanic males and 41.6 % among adolescent Hispanic females [10, 12], both substantially below the Healthy People 2020 target of 80 % coverage [46]. In one study that examined the effect of a HPV fact sheet among female college students, the intervention resulted in only a 5.5 % HPV vaccine uptake rate despite a 41 % baseline intent rate [47]. The discrepancy between acceptance and uptake could be due to institutional barriers to immunization access or social desirability bias in the survey.

Character identification has previously been described as a mechanism by which narratives influence behavior. In a narrative-based sexual health intervention, Moyer-Gusé et al. showed that identification with role model characters motivated participants to engage in sexual health discussions by increasing self-efficacy [48]. In another study, Frank et al. investigated the role of character identification in a HPV narrative film. The researchers found that participants who identified with characters were more likely to perceive higher susceptibility to HPV, but less likely to perceive its severity [28]. In the present study, participants younger than 21 years and those older than 23 years identified with the characters, but only the younger participants were willing to vaccinate and to encourage their friends and family to vaccinate after exposure to the fotonovela. This finding brings up the possibility of other elements of narrative processing that may be important for changing behavior. Determining which narrative elements are most influential for changing attitudes and behaviors will allow us to hone in on certain parts of the intervention for maximum effectiveness.

To our knowledge, there is no other narrative-based HPV vaccination educational intervention that target Hispanic young adults; existing interventions have focused on Hispanic parents [27–29]. The rationale for tailoring to the young adult population is twofold. First, large proportions of males and females in this age group remain unvaccinated [11]. Second, young adults are candidates for the vaccine themselves, but they could also be parents of children who are faced with decisions to vaccinate. Overall, nearly all the young adult participants in the present study viewed the fotonovela favorably. Hispanic and non-Hispanic participants alike found the intervention to be entertaining, informative, and relevant.

The results of this study should be interpreted in light of several limitations. This study used a pre-experimental design as a cost-effective and exploratory approach to examine the effectiveness of our fotonovela intervention. An important drawback is the potential threat to internal validity due to the lack of a control group. Another limitation was the small sample size. Due to constraints in resources, the study was underpowered, and thus, may not have detected the actual effects of the fotonovela. Some participant responses were collapsed into a broader category in order to correct for the relatively small sample size. While this allows for more meaningful analyses, some data was inevitably lost during this process.

Whether or not participants were vaccinated may influence their knowledge, attitudes and intentions to the vaccine. Unfortunately, HPV vaccination status was not collected in this study, although such information would have been particularly informative for interpreting their perceptions to HPV susceptibility. We do not know whether the low reported perceived susceptibility of HPV was due to feeling protected from already having received the vaccine or truly believing they were not at risk of HPV. During data collection, three participants voluntarily reported that they received the vaccine, and evidently, perceived themselves to be at low risk of infection. However, without knowledge of other participants’ vaccination status, we cannot infer whether or not the low perceived susceptibility was related to prior vaccination. All participants, regardless of reported vaccination status, were included in analyses because this information should not influence their attitudes towards the fotonovela.

A major advantage of the study was the inclusion of Hispanics and non-Hispanics, males and females. However, the overwhelming majority of participants were Hispanic women due to the women’s health clinic at UCI-FHC. Of the men who refused to participate, the predominant reasons were lack of interest in research topic and in incentive offered. The fotonovela was written at a sixth-grade reading level and intended for readers with minimal health literacy. However, most of the participants achieved at least a high school education and thus, may be more health literate than Hispanics who did not complete high school. While the 13 % of participants who did not attain a high school diploma still found the fotonovela easy to understand, it cannot be known with certainty whether this particular intervention would be suitable for everyone. Future research is needed to demonstrate generalizability and corroborate the appropriateness of the fotonovela within a larger sample of individuals across various cultures, educational levels, socioeconomics, and age groups, including adolescents. Lastly, because this study only examined changes in knowledge, attitudes and intentions related to vaccination immediately post-intervention, it would be worthwhile to study the fotonovela’s long-term impact, particularly in vaccination behavior.

## Conclusion

The fotonovela “What You Don’t Know” distinguishes itself as a unique educational tool. It is one of the first culturally tailored educational interventions designed to promote HPV vaccine acceptance among Hispanic young adults. Findings from this study demonstrated the utility of the fotonovela in impacting HPV vaccine knowledge, attitudes, and intentions. Implications of the fotonovela are enormous. The fotonovela can be used by healthcare providers to engage patients into dialogues about HPV vaccination, distributed within social networks to reach people who may not be seeking this information, incorporated into school curricula as an educational tool for sexual health, and used to empower individuals to take preventive actions in their personal health as well as their children’s health. Future efforts should examine the effectiveness of the fotonovela in conjunction with other educational interventions and explore alternative avenues and media outlets to disseminate the fotonovela to broaden its reach to new audiences.
